# Supplementary material for: Cavity-enhanced single artificial atoms in silicon
Source: Nat Commun. 2024 Jun 21;15:5296. doi: 10.1038/s41467-024-49302-0 (PMC11192735; doi:10.1038/s41467-024-49302-0)
Supplement: Supplementary file 1 — Supplementary Information [file 41467_2024_49302_MOESM1_ESM.pdf]

SUPPLEMENTARY INFORMATION: CAVITY-ENHANCED SINGLE ARTIFICIAL ATOMS IN SILICON

Valeria Saggio[1], Carlos Errando-Herranz[1,2], Samuel Gyger[1,3], Christopher Panuski[1], Mihika Prabhu[1], Lorenzo De Santis[1,4], Ian Christen[1], Dalia Ornelas-Huerta[1], Hamza Raniwala[1], Connor Gerlach[1], Marco Colangelo[1], and Dirk Englund[1]

[1]Massachusetts Institute of Technology, Cambridge, MA, USA
[2]University of Münster, Münster, Germany
[3]KTH Royal Institute of Technology, Stockholm, Sweden
[4]QuTech, Delft University of Technology, Delft, The Netherlands

1. Cavity optimization

Guided mode expansion (GME) allows the eignfrequency and eigenmode of a slab photonic crystal (PhC) cavity to be accurately and efficiently estimated using the incomplete basis set of guided modes of an unpatterned effective slab [1, 2]. Fig. S1 shows a sample GME simulation supercell of dimensions $L_x \times L_y$ for a small-mode volume L4/3 PhC cavity [3]. The cavity quality factor Q can also be estimated with GME by computing the perturbative coupling between the eigenmode and the continuum of radiative modes of the unpatterned slab. Previous work [2, 4] has therefore focused on optimizing Q as a function of the hole shifts ($\delta\vec{x}$, $\delta\vec{y}$) and/or radii perturbations $\delta\vec{r}$, yielding optimized designs similar to that of Fig. S1. The quality factor improvement often comes at the expense of coupling efficiency, as the leakage from Q-optimized cavities typically occurs at large emission angles.

We ultimately desire a cavity with both high-Q and efficient vertical beaming. Fortunately, automatic differentiation allows the gradient of any objective function based on the GME outputs to be efficiently computed with respect to the input parameters. We have previously shown that the GME outputs can accurately approximate the cavity's far-field profile, thereby enabling optimization via automatic differentiation [5]. The general technique is illustrated in Fig. S2. The radiative quality factors in GME are computed via first order perturbation theory, which lends the coupling coefficients $c_{ij}^{s,p}$ between the cavity mode and the diffraction order associated with the reciprocal lattice vector $\vec{G}_{ij}$ for s (TE) and p (TM) polarizations [2]. Since GME assumes periodic boundary conditions for a desired in-plane wave vector $\vec{k}$, each coefficient corresponds to emission in the direction $\vec{d}_{ij}(\vec{k}) = (\vec{k} + \vec{G}_{ij})/k$ in sine space — $(u, v) = (\sin\theta\cos\phi, \sin\theta\sin\phi)$ for the zenith and azimuth angles θ and ϕ, respectively — given the cladding wave-number $k = 2\pi/\lambda$. A single GME simulation therefore approximates the radiative intensity $S_{ij}(\vec{k}) = |c_{ij}^s(\vec{k})|^2 + |c_{ij}^p(\vec{k})|^2$ for all diffraction orders within the light cone ($|\vec{d}_{ij}(\vec{k})| < 1$). With a sufficiently large supercell of dimension $(L_x, L_y) \gg a$, the small magnitude of principal reciprocal lattice vectors ($|\vec{G}_{1,0}|$, $|\vec{G}_{0,1}|$) $\ll k$ allows a coarse far-field emission pattern to be reconstructed. Higher far-field resolution is then possible by running the GME simulation with multiple $\vec{k}$ within the Brillouin

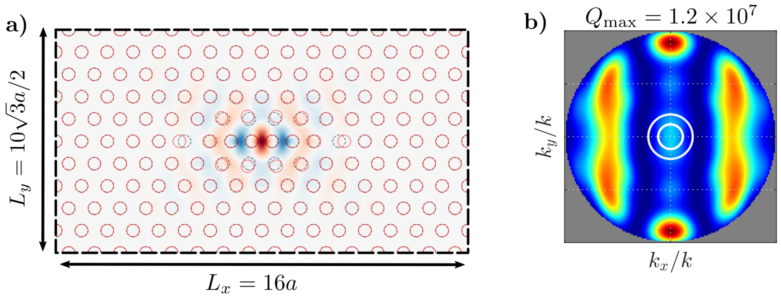

Figure S1. **Q-optimized cavity design with guided mode expansion.** GME design cell for an air-clad L4/3 silicon PhC cavity in a 220 nm-thick silicon membrane with lattice constant $a = 420$ nm and default hole radius $r/a = 0.238$. Given hole displacement and scale vectors $\delta\vec{x}$, $\delta\vec{y}$, $\delta\vec{r}$, the cavity mode and quality factor are approximated at desired in-plane wave-vectors $\{\vec{k}\}$ set by periodic boundary conditions (dashed black box). Red holes in (a) correspond to the Q-optimized hole locations, which produce the finite difference time domain (FDTD)-computed far-field profile and maximum quality factor $Q_{\max}$ in (b). White circles correspond to the diffraction-limited beamwidths $\theta \sim \lambda/Lx$, λ/L_y.

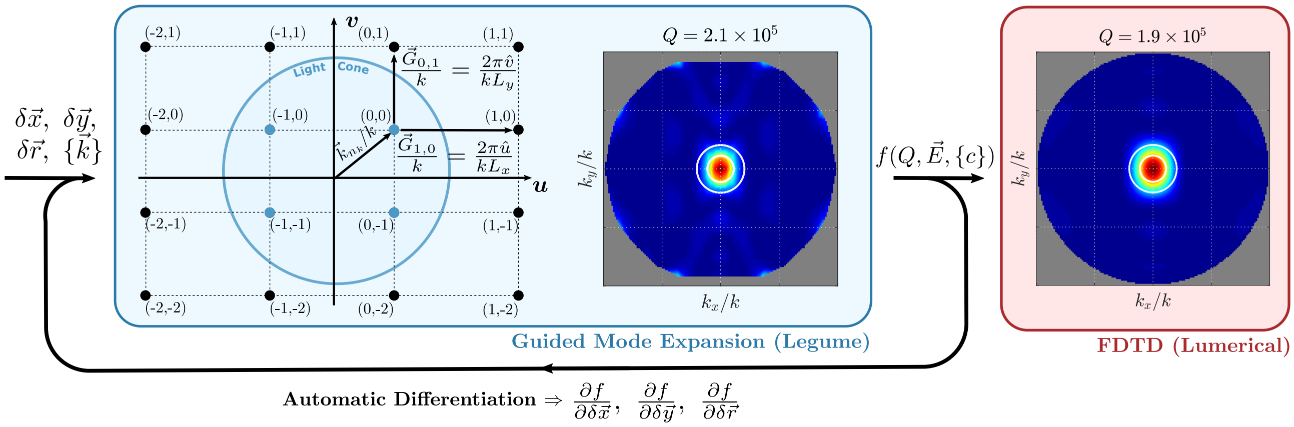

Figure S2. **Overview and results of the far-field optimization process for the L4/3 PhC cavity in Fig. S1.** Given displacement and scale vectors ($\delta\vec{x}$, $\delta\vec{y}$, $\delta\vec{r}$), the cavity near-field and quality factor are approximated at a desired $\vec{k}$ (which is set by Bloch periodic boundary conditions) with `Legume`, an open-source guided mode expansion (GME) package [2]. The far-field emission into diffractive orders within the light cone — located at $(\vec{k}+\vec{G}_{ij})/k$ for the simulated $\vec{k}$, the reciprocal lattice vectors $\vec{G}_{ij}$ of the rectangular supercell lattice, and resonant mode wave-number $k = 2\pi/\lambda$ in the device cladding — can also be approximated using the perturbation theory-computed coupling coefficients $\{c\}$ between the cavity mode and radiative continuum of an unpatterned slab. Multiple $\vec{k}$ can be simulated to increase the resolution of the approximated far-field profile. An objective function f based on the Q, near-field $\vec{E}$, and far-field profile derived from $\{c\}$ can then be efficiently optimized using automatic differentiation. Optimizing the geometry in Fig. S1 with a simulation resolution $g_{\max} = 2.5 \times 2\pi/a$ and two $\vec{k}$ yields the depicted GME far-field pattern when verified with $g_{\max} = 3 \times 2\pi/a$ and 25 simulated $\vec{k}$. The GME result is validated with FDTD simulations using a conformal mesh size $a/20$, which confirm the near-diffraction-limited far-field profiles.

zone and aggregating the coupling coefficients.

Using the GME results, we compute an optimization objective function

$$f = \underbrace{\frac{S_{0,0}(0)}{\sum_{i,j,n_k} S_{ij}(\vec{k}_{n_k})}}_{\eta_0} \arctan\left(\frac{Q}{Q_{\text{design}}}\right) |E_y(0,0)|^2 \tag{S1}$$

intended to: 1) maximize the zero-order diffraction efficiency η_0, the ratio of normal radiative emission $S_{0,0}(0)$ to that summed over all diffraction orders and simulated $\vec{k}$; 2) increase Q to a set design value Q_{design}; and 3) localize the near-field (thereby minimizing the mode volume) by maximizing the center-plane electric field $E_y(x,y)$ at the center of the cavity. We initially considered optimization with a simpler objective function $f = \eta_0 Q$, but encountered two problems. First, the simplified objective yielded Qs near the maximum value $Q_{\max}$ achievable for the simulation resolution $g_{\max}$, the maximum reciprocal lattice wave-vector magnitude in the truncated GME basis set. The resulting far-field profiles varied as a function of $g_{\max}$, indicating that the far-field emission profile was dominated by resolution-related losses. By replacing $Q \to \arctan(Q/Q_{\text{design}})$ in the objective, the optimized Q approaches Q_{design}, yielding the far-field pattern invariant to changes in $g_{\max}$ if $Q_{\text{design}} \ll Q_{\max}$. In addition, the simplified objective produced near-field profiles extending to the edge of the simulation supercell, leading to undesired coupling between adjacent cavities (when using arrays). Including a factor ($|E_y(0,0)|^2$) inversely proportional to the Purcell mode volume solved this problem and produced well-localized modes at the center of the cavity.

The gradient of the final optimization objective function of Eq. S1 can then be efficiently computed with respect to every design parameter using automatic differentiation. For example, we optimized the L4/3 hole locations with $Q_{\text{design}} = 5 \times 10^4$ to find the GME-computed far-field and Q (2.1×10^5) shown in Fig. S2. These results compare favorably with first-principles finite-difference time-domain (FDTD) simulations: the emission patterns are qualitatively similar and the Qs match within 10%. As desired, the localization of surface-normal cavity leakage is significantly improved from that of the Q-optimized cavity shown in Fig. S1b.

Figure S3 shows the course of optimization for the two cavities we employed in our experiments, with the only difference being the hole sizes. Sweeps with several varying parameters (e.g. lattice constant, cavity and hole size) were fabricated in order to target systems with the desired performance.

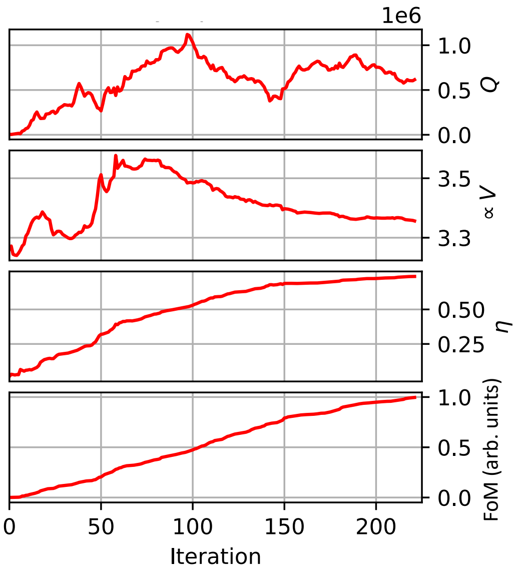

Figure S3. **2D photonic crystal cavity optimization path.** The course of optimization for one of our designs over 222 iterations, plotting the simulated quality factor Q, a result proportional to the mode volume V, the zero-order diffraction efficiency η_0, and the composite figure of merit FoM targeted by the optimization.

2. Measurement setup

Fig. S4 shows a schematic of our measurement setup, which consists of 3 main optical paths: 1) excitation, 2) cryogenic 4F, and 3) collection.

The excitation path combines IR and visible collimated lasers with a dichroic, and routes them via a linear polarizer into a polarizing beam splitter (PBS). For our characterization, we use two visible lasers and two infrared lasers. Our visible lasers consist of a continuous wave Coherent Verdi G5 at 532 nm, and a pulsed laser from NKT Photonics (SuperK) with a maximum repetition rate of 78 MHz and filtered by a bandpass filter centered at 532 nm with a bandwidth of 0.2 nm. Our infrared lasers are a tunable CW O-band TSL570C from Santec, and a superluminescent diode S5FC1018S from Thorlabs (operating at 600 mA) with a broadband emission centered at 1310 nm, both working at room temperature. The IR and visible beam sizes are $\approx$ 3.5 mm before the 4F system, to match the objective's pupil diameter. A set of scanning mirrors is placed in the excitation path for precise beam positioning and PL mapping of our cavities.

The transmitted polarization component is imaged into our cryostat by using a 4F lens system consisting of scanning piezoelectric mirrors, two lenses, and an objective, together with IR quarter and half wave plates for polarization rotation. Both wave plates were used to obtain the polarization plot in Fig. 3a. Our microscope objective is a collar corrected objective LCPLN50XIR from Olympus with a NA of 0.65, and is external to the cryostat. Our cryostat is a Montana Instruments system. The sample is mounted on a XYZ cryogenic piezoelectric stages (Attocube). As these stages exhibit limited thermal conductivity, thermal links between the cold finger (placed beneath the stage) and the sample (mounted on top of the stage) are used to ensure proper thermalization of the sample.

The PL and reflection from our sample are collected via the PBS reflection through an IR polarizer and a filtering station into a fiber switch, which routes the collected light into superconducting nanowire single-photon detectors (SNSPDs) or an IR spectrometer. Our filtering setup consists of a longpass filter (cutoff wavelength at 1250 nm) and a shortpass filter (cutoff wavelength at 1300 nm). Additionally, we used a tunable fiber filter from WL Photonics with FWHM transmission bandwidth of 0.10 nm to perform lifetime measurements and gas-tuned second-order correlation measurements on closely cavity-coupled emitters. Our two SNSPDs (NIST) feature detection efficiencies of 21% and 24%, and are readout with a Swabian Instruments Timetagger 20. Our IR spectrometer consists of a PyLon IR CCD from Princeton Instruments and switchable gratings, one with a density of 300 gr/mm and a 1.2 µm blaze and another with a density of 900 gr/mm and a 1.3 µm blaze, leading to pixel-defined resolutions of 155 pm and 40 pm respectively. For the second-order autocorrelation measurements we used a fiber beam splitter (Thorlabs TW1300R5F1) after the filtering station. In addition, we imaged our sample using a flip mirror before the fiber switch and an InGaAs cooled CCD camera (Allied

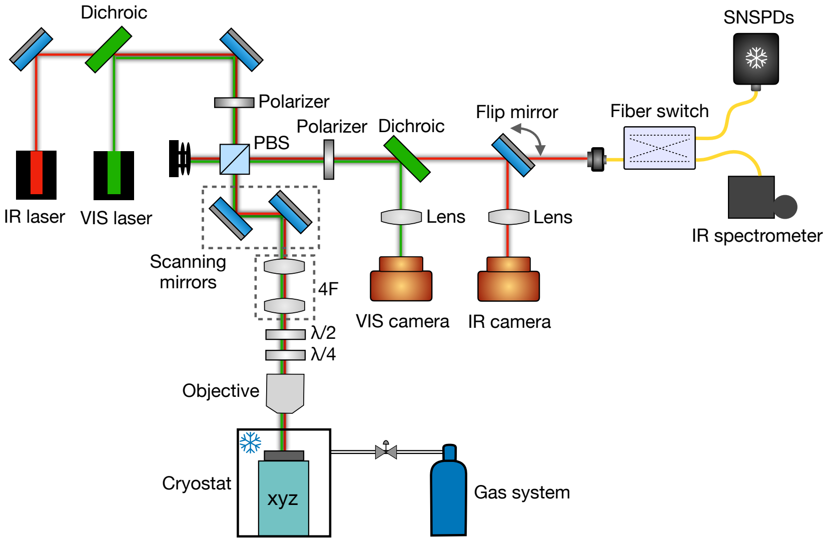

Figure S4. **Measurement setup schematic.** Our setup consists of a cryogenic confocal microscope. Laser light at visible (532 nm) and infrared (1280 nm) shines through a polarizer and a polarizing beam splitter (PBS), a set of galvanometers (scanning mirrors), a 4F system, and polarization rotation components, into an objective and into the cryostat. The IR PL or cavity reflectivity from the sample reflects off the PBS through a polarizer and a filtering stage into the fiber switch, which leads to SNSPDs or an IR spectrometer. A dichroic enables imaging of the visible light on a visible camera. A flip mirror and a lens between the dichroic and the fiber switch enable the visualization of the image plane into an IR camera, and taking away the lens provides us with a back focal plane image. The cryostat is equipped with a gas line system for controllable gas injection.

Vision Goldeye), preceded by a flip lens that allows us to switch between near-field and far-field imaging. To enable alignment of the visible and IR beams, we used a visible camera (Thorlabs Zelux) to image the visible light.

The setup comprises also a gas line equipped with a needle valve and several ball valves to enable controllable gas injection into our cryostat.

In our experiment, one sample hosting thousands of cavities — each nominally different from all others — was used. After targeting a few cavities (∼10) based on Q-factor, size and resonance wavelength, each of them was probed to look for the presence of a single emitter. Two of them were then chosen and analyzed. We discarded data sets that did not show the presence of an emitter, or that revealed multiple ones.

3. Cross-polarization cavity characterization

We characterized our cavities via a cross-polarization measurement, as previously reported in Ref. [6]. The measurement protocol consists of preparing the polarization of the input and output IR beams to be orthogonal and 45° rotated with respect to the cavity axis. This was achieved by setting the input and output polarizers perpendicular to each other, and by rotating the common half-wave plate to align the fields to the cavity (see schematic in Fig. S4). In our setup, we used a PBS for increased polarization extinction.

4. Measurements under pulsed excitation

All measurements under pulsed excitation were performed with our SuperK laser using a repetition rate of 39 MHz, and varying the power from a minimum of 0.2 µW to a maximum of 2 µW.

As already discussed in the main text, our artificial atom emission fits well to the characteristic two-level emitter saturation model, which is given by

$$I(P) = I_{\infty} \frac{P}{P + P_{\text{sat}}}. \tag{S2}$$

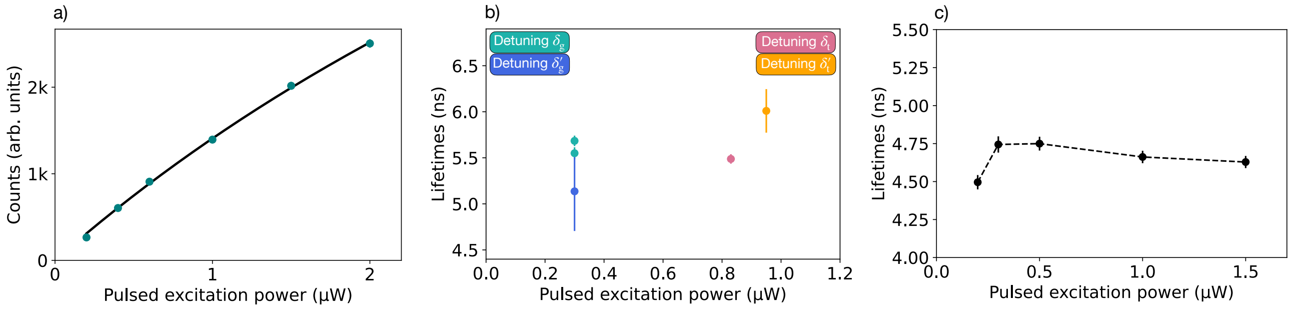

Figure S5. **Measurements under pulsed excitation**. a) Saturation curve obtained acquiring PL counts while varying the pulsed excitation power. b) Lifetimes displayed both for the gas and thermal tuning cases. In each case, we measured the lifetimes at similar pulsed excitation powers. c) Set of lifetime measurements collected at detuning δ_g under possibly different laser conditions. In both plots b) and c), the error bars are the errors extracted from the lifetime fits.

Here, I and P are intensity and power, respectively, and I_∞ and P_{sat} are the corresponding saturation values. Fitting our experimental data to this theoretical model, we find $I_\infty = (12 \pm 2)$ Kcounts/min and $P_{sat} = (7.4 \pm 1.2)$ μW. The data and corresponding fit are shown in Fig. S5a. The same theoretical model is used to fit the data taken under CW excitation power reported in the main text. In that case (Fig. 3b in the main text), we obtain $I_\infty^{CW} = (93 \pm 5)$ Kcounts/min and $P_{sat}^{CW} = (28 \pm 3)$ μW. As already mentioned in the main text, the PL counts reported in the saturation curves are extracted from spectroscopy measurements using a grating density of 300 gr/mm.

Fig. S5b shows the excited state lifetime values for all the detunings. In the gas tuning case, two measurements were taken under the same power and detuning. In both thermal and gas tuning cases, the lifetime does not differ significantly for different detunings. The single value shown in Fig. 3f in the main text for detuning δ_g is the weighted average between the two values reported in Fig. S5b. For the sake of completeness, we also report additional lifetimes acquired at detuning δ_g under likely different laser conditions, originating from an error related to our laser control electronics board. The values are shown in Fig. S5c. In this case, we measure lifetimes all below 4.75 ns. As these measurements were taken under possibly different experimental conditions, we decided not to include them in our theoretical analysis. However, they confirm that the excited state lifetime of our emitter remains essentially unchanged with increasing excitation power. All other reported lifetimes were

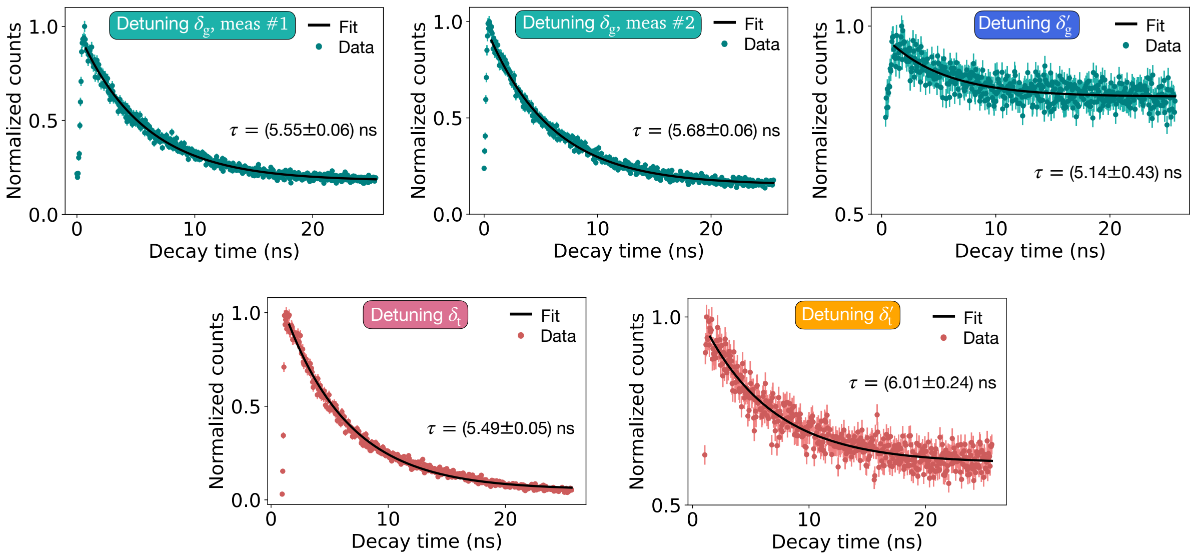

Figure S6. **Lifetime data**. Raw lifetime data and fits shown for both gas and thermal tuning cases under different detunings δ_g, δ_g', δ_t, and δ_t'. Two measurements were acquired at detunings δ_g. Poissonian error bars are shown in all plots. The errors in the lifetime values are extracted from the fits.

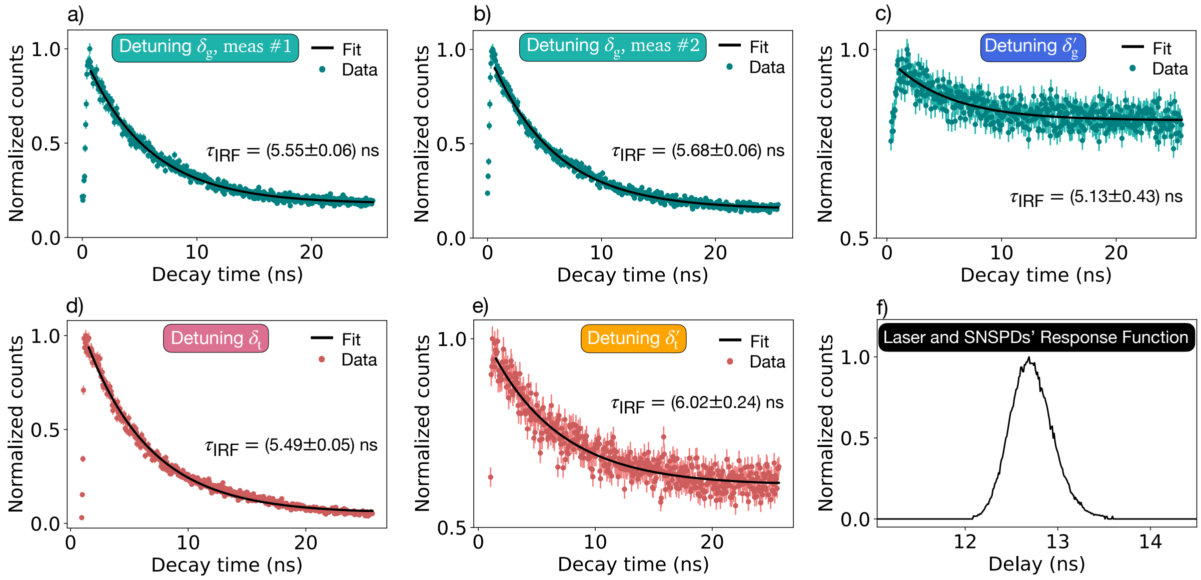

Figure S7. **IRF-corrected lifetime measurements**. a-e) Lifetime data and IRF-corrected fits. f) Laser and SNSPDs' response function.

instead taken under the same laser conditions, which ensures the correctness of our lifetime comparisons. All lifetime data sets are fitted to a mono-exponential model

$$f(t) = x\ e^{-\frac{t-y}{\tau}} + z, \tag{S3}$$

to extract the excited state lifetime τ. x, y, and z are additional fitting parameters. All fits and extracted values used in the analysis are reported in Fig. S6 for the different detuning cases.

We note that the lifetime data presented in Fig. S6 are not corrected for the laser and SNSPDs' response function. In general, it is important to take the Instrument Response Function (IRF) into account, as it may distort the signal and thus affect the reliability of the fits. For this reason, we measured the IRF and convolved it with the fit function in Eq. S3. In this way, we properly include the laser and SNSPDs' response when extracting our lifetime values. The IRF-corrected data are displayed in Fig. S7, together with the IRF curve. The extracted lifetime values do not vary significantly in comparison to the previous case where no IRF correction was applied.

5. Second-order autocorrelation measurements

The second-order autocorrelation measurements were performed using a HBT interferometer. We excited each of our emitters — when closely coupled to their cavities — with a 532 nm CW pump and sent the generated photons to a fiber beam splitter whose outputs were connected to two SNSPDs, and we then analyzed the coincidence counts at different time delays between the two outputs. Fitting our second-order autocorrelation data with a three-level system equation

$$g^{(2)}(t) = a\left[1-(1-b)\left((1+c)e^{-\frac{|t-t_{\mathrm{shift}}|}{\tau_1}} - c\ e^{-\frac{|t-t_{\mathrm{shift}}|}{\tau_2}}\right)\right], \tag{S4}$$

with a, b, and c fitting parameters, t_{shift} the offset for the time delay t, and τ_1 and τ_2 the lifetimes, we obtain the $g^{(2)}(0)$ value after data normalization as $g^{(2)}(0) = b$ at $t = t_{\mathrm{shift}} = 0$. HBT measurement data and fits are shown in Figs. S8a and b for the thermal tuning and gas tuning cases, respectively. In both cases we obtain a $g^{(2)}(0)$ close to 0, thus confirming genuine single-photon emission. In the gas tuning case, we find $g^{(2)}(0)^{\mathrm{g}} = 0.03^{+0.07}_{-0.03}$, $\tau_1^{\mathrm{g}} = (3.16 \pm 0.5)$ ns and $\tau_2^{\mathrm{g}} = (9.05 \pm 1.3)$ ns. We stress here that $g^{(2)}(t)$ measurements at different powers and subsequent extrapolation of the decay rate from the excited to the ground state would be needed to obtain

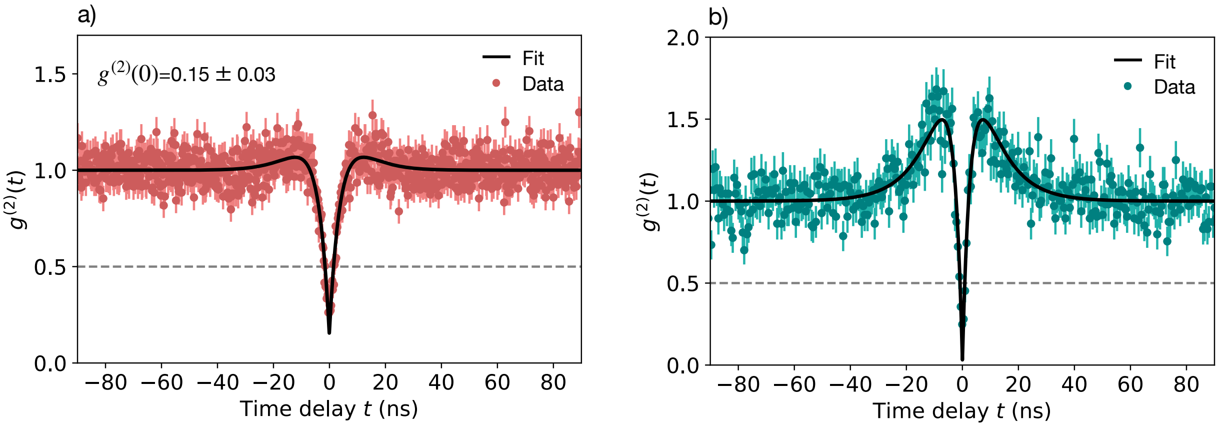

Figure S8. **Second-order autocorrelation measurements.** a) HBT measurement result from the thermally tuned cavity-atom system, and b) zoomed out version of the gas tuning cavity-atom system shown in Fig. 3d. Both measurements show clear $g^{(2)}(0) \approx 0$, demonstrating single-photon emission. In both measurements, poissonian error bars are included for each data point, and the error in the $g^{(2)}(0)$ is extracted from the fit.

reliable lifetime values [7–9]. Hence, the lifetimes extracted from this fit are not to be considered an indication of the actual values. We only perform $g^{(2)}(t)$ measurements to obtain information about the nature of the emission. To extract reliable lifetime values, we perform pulsed laser measurements instead. The $g^{(2)}(t)$ data was collected filtering the region around the ZPL, and thus reducing the noise contribution coming from elsewhere. In the thermal tuning case, we used the longpass and shortpass filters to isolate a $\sim$ 50 nm-wide region comprising the ZPL, while we improved the gas-tuning measurement by filtering a much narrower region with the only help of the electrically tunable bandpass fiber filter described in Sec. 2. We used a CW excitation power of 6 µW (10 µW) in the thermal (gas) tuning case. In both cases the data was acquired as raw time tags and is not background-corrected. The coincidence counts were evaluated with the software ETA [10] using a time binning of 200 ps (512 ps) in the thermal (gas) tuning case.

The G-center is expected to feature three energy levels: a ground and excited singlet state and a metastable triplet state [11]. The metastable state may introduce a bunching effect in the second-order correlation, and has been previously observed in related works [7, 8]. This bunching effect is clearly visible in Fig. S8b, while it is less evident in Fig. S8a. We attribute this difference to the fact that the two measurements were taken at different optical powers, and it is therefore not straightforward to make a direct comparison. Moreover, different emitters have different electron trapping rates and other mesoscopic properties that dictate the bunching. Although the presence of a metastable state would explain the observed bunching and is in line with what formulated in previous works [7, 8], we would like to stress that others factors such as spectral diffusion may play a role in dictating the bunching properties.

6. Tuning of cavity resonances

Here, we expand on the methods used to decouple the G-centers from our cavities. As mentioned in the main text, we performed both thermal and gas tuning to achieve decoupling.

a. *Thermal tuning*

Starting with a cryostat chamber temperature of $\sim$ 4 K, we define the detuning δ_t as $\delta_t = \lambda^t_{cav} - \lambda^t_{ZPL}$, where λ^t_{ZPL} is the G-center ZPL wavelength and λ^t_{cav} the cavity resonance wavelength. Fitting both the cavity and ZPL profiles to a Lorentzian function, we find the wavelengths to be $\lambda^t_{cav} = (1279.747 \pm 0.002)$ nm and $\lambda^t_{ZPL} = (1279.850 \pm 0.004)$ nm, and thus calculate $\delta_t = (-0.103 \pm 0.004)$ nm. The cavity and ZPL profiles at $\sim$ 4 K are displayed in pink in Fig. S9. The extracted Q factor of our cavity is 3725 ± 50.

In order to decouple the cavity-atom system, we warmed up the chamber to $\sim$ 24 K and observed the subsequent effect. As visible in orange in Fig. S9, the cavity shifted away spectrally from the ZPL, resulting

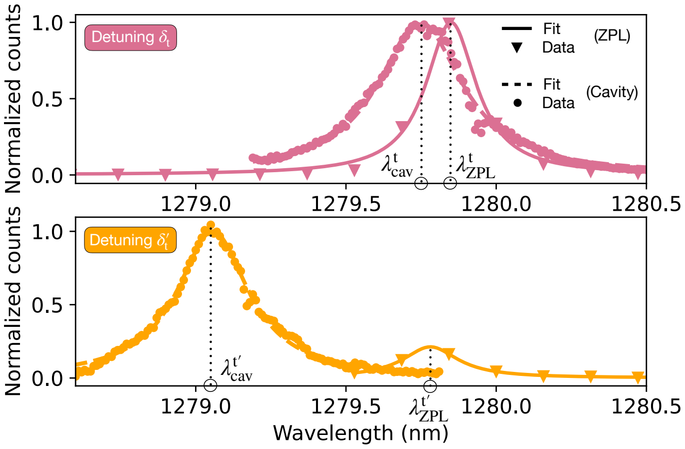

Figure S9. **Thermal tuning of a cavity.** Decoupling of the cavity-atom system is achieved by changing the temperature in the cryostat chamber from ~ 4 K to ~ 24 K, leading to a detuning of $\delta_{\rm t}$ (top) and $\delta_{\rm t}'$ (bottom), respectively. A significant cavity wavelength shift — from $\lambda^{\rm t}_{\rm cav}$ to $\lambda^{\rm t'}_{\rm cav}$ — as well as a much less evident emitter ZPL shift — from $\lambda^{\rm t}_{\rm ZPL}$ to $\lambda^{\rm t'}_{\rm ZPL}$ — are observed.

in a reduction of the ZPL intensity. The cavity resonance is now found at $\lambda^{\rm t'}_{\rm cav} = (1279.057 \pm 0.001)$ nm, and a less significant spectral shift is also observed in the ZPL wavelength, now at $\lambda^{\rm t'}_{\rm ZPL} = (1279.781 \pm 0.001)$ nm. The latter effect is in line with what we recently reported in Ref. [12], where a non-volatile spectral shift in the ZPL of our single G-centers was observed. After the temperature variation, the detuning is found to be $\delta_{\rm t}' = (-0.724 \pm 0.001)$ nm. Fig. 3d in the main text condenses this information by combining both detuning cases $\delta_{\rm t}$ and $\delta_{\rm t}'$ in one plot. To highlight these detunings between the cavity and ZPL, we shifted the x-axis of the bottom plot in Fig. S9 by ($\lambda^{\rm t}_{\rm ZPL}$ - $\lambda^{\rm t'}_{\rm ZPL}$). In this way, the detunings between the cavity and ZPL are more clearly visible. However, because of this shift, it should be noted that the plot in Fig. 3d does not reflect the real difference between the cavity wavelengths before and after tuning. This information is preserved in Fig. S9.

All spectra were acquired with a grating density of 300 gr/mm. The cavity reflectivity measurements were performed with our tunable CW narrowband IR laser while sweeping its wavelength and recording the cavity spectrum at each step. All spectra were then merged to obtain the complete cavity profile.

b. Gas tuning

To validate what we observed in the thermal tuning case, we spectrally shifted the cavity of a second cavity-atom system via a different tuning mechanism based on gas deposition and subsequent sublimation. In this case, the chamber temperature remained constant (at ~ 8 K) throughout our measurements. First, we coated our sample with a thin layer of gas (CO_2) by injecting the gas into our cryostat chamber via a dedicated gas line. The gas layer alters the mode index of the cavity and thus redshifts its wavelength. We assume first-order perturbation theory and follow the derivation in the SI of Ref. [5]. Using a refractive index for solid CO_2 of 1.4 [13], and a representative mode for the photonic crystal cavity, we estimate a resonance shift of the order of 10 nm for an infinite thickness of solid CO_2.

We then performed gradual gas sublimation to achieve a controllable cavity resonance blueshift. Fig. S10a shows a representative experimental gas shifting of cavity spectra. Our maximum tuning, in the order of 1 nm, aligns reasonably well with our theoretical estimate. Starting from the nominal cavity resonance wavelength (purple curve), we injected CO_2 and thus redshifted the cavity mode (black curve). We then illuminated the cavity with a 532 nm CW pump for ~ 0.5 s at increasingly higher powers (displayed in the figure's legend), and thus achieved a controllable cavity resonance shift. We note that our cavity blueshifts further than its initial central wavelength, indicating that there is likely a significant leakage of CO_2 or other gases during the cooling, which we can sublimate using our optical pumping technique. CO_2 deposition results in the cavities' resonances remaining extremely stable for weeks.

After depositing CO_2 on our sample, we acquired the ZPL and cavity spectra. We find their center wavelengths to be $\lambda^{\rm g}_{\rm ZPL} = (1279.277 \pm 0.001)$ nm and $\lambda^{\rm g}_{\rm cav} = (1279.354 \pm 0.002)$ nm, leading to a very small detuning

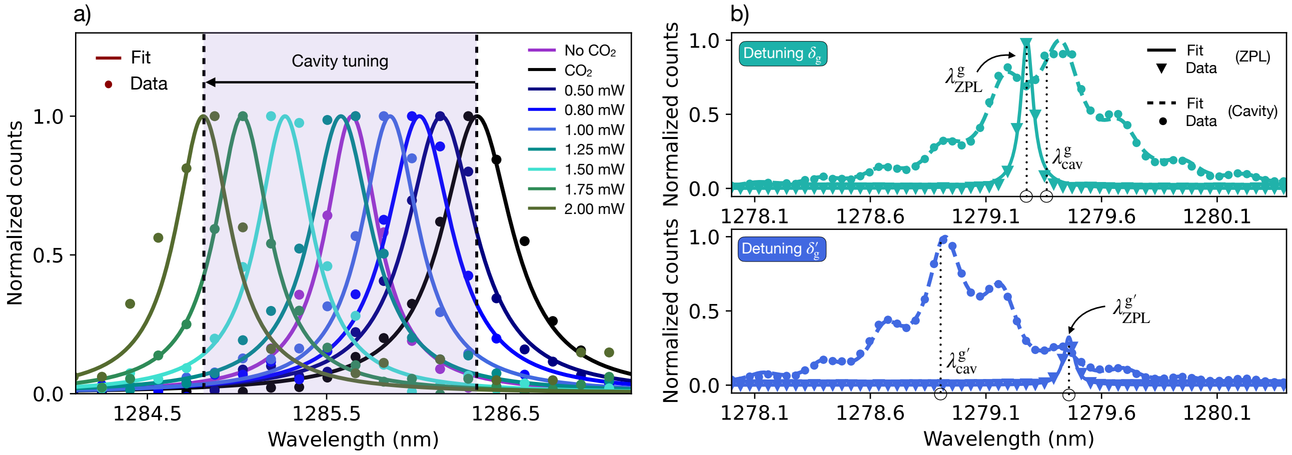

Figure S10. **In-situ gas tuning of a cavity.** a) Starting from its nominal wavelength (center of purple curve), the cavity resonance is redshifted by introducing CO_2, and controllably blueshifted by sequentially burning gas using a 532 nm CW pump at different powers (indicated in the legend). b) Decoupling of the emitter from the cavity using this gas tuning method. The figure shows the ZPL and cavity profiles under different detunings δ_g (before gas tuning, top) and δ'_g (after gas tuning, bottom). A cavity wavelength shift — from λ^{g}_{cav} to $\lambda^{g'}_{cav}$ — as well as a ZPL shift — from λ^{g}_{ZPL} to $\lambda^{g'}_{ZPL}$ — are observed.

of $\delta_g = \lambda^{g}_{cav} - \lambda^{g}_{ZPL} = (0.077 \pm 0.002)$ nm as shown in green in Fig. S10b. Unlike the previous case, the cavity reflectivity measurements shown in Fig. S10b were performed with our superluminescent diode by simply illuminating the cavity and recording its reflectivity spectrum. Moreover, these spectra were acquired with a higher resolution compared to the previous experiments (here we used a grating density of 900 gr/mm) which resulted in a slight wavelength offset compared to the 300 gr/mm case, likely due to calibration errors. This offset was taken into account when plotting the data, in order to enable a fair wavelength comparison between the thermal and gas tuning cases.

While the value of λ^{g}_{ZPL} was still obtained by fitting the ZPL profile to a Lorentzian function, the cavity spectrum required a different analysis due to the presence of parasitic oscillations in its profile, as visible in Fig. S10b. This behaviour was not observed in the thermal tuning case because of the lower resolution arising from a smaller grating density. It is known that these parasitic cavities are Fabry-Pérot (FP) cavities arising from reflections in the silicon substrate [5]. A FP cavity is described by an airy distribution [14] as

$$f_{FP} = \frac{A(1-R_1)^2 R_2}{(1-\sqrt{R_1 R_2})^2 + 4\sqrt{R_1 R_2}\sin^2\phi}, \tag{S5}$$

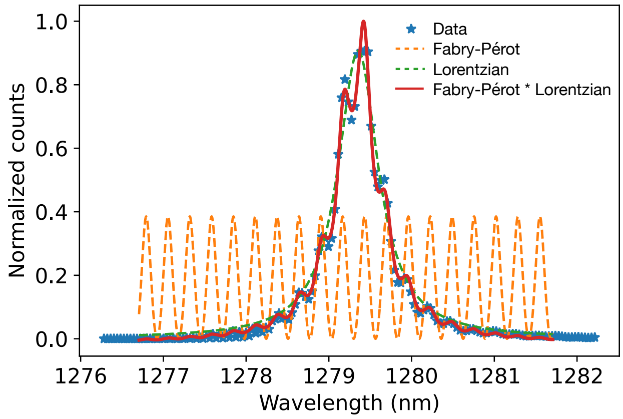

Figure S11. **Cavity resonance fit.** The combined Airy-Lorentzian (red) fits well our cavity reflectivity measurements (blue stars), and can be decomposed in a Fabry-Pérot cavity (orange dashed) and a Lorentzian (green dashed).

with A an amplitude fitting constant, $\phi = \pi(v - v_0)/v_{\mathrm{FSR}}$, $v - v_0$ the optical frequency detuning, $v_{\mathrm{FSR}} = c/2L$ the cavity free spectral range with c the speed of light and L the cavity length, and R_1 and R_2 the mirror reflectivities.

The photonic crystal cavity resonance is described by a Lorentzian as follows

$$f_{\mathrm{L}} = \frac{B}{(\lambda - \lambda_0)^2 + (\Gamma/2)^2} + C, \tag{S6}$$

with B and C being fitting constants for the amplitude and offset, λ and λ_0 the wavelength and wavelength offset, and Γ the full width at half maximum (FWHM). We estimate the effect of the coupled system by using a product function

$$f_{\mathrm{tot}} = f_{\mathrm{FP}} f_{\mathrm{L}}. \tag{S7}$$

The fit including these parasitic oscillations is displayed in Fig. S11. We extract a cavity Q factor of 2136 ± 30.

To decouple our cavity-atom system, we illuminated a region in the vicinity of our cavity with 532 nm CW laser light with powers up to 550 μW. This resulted in a shift of our cavity resonance wavelength from $\lambda^{\mathrm{g}}_{\mathrm{cav}}$ to $\lambda^{\mathrm{g}'}_{\mathrm{cav}} = (1278.976 \pm 0.001)$ nm. We note that also in this case the optical pumping required for gas tuning introduces a non-volatile spectral shift in the ZPL of our single emitters, as reported in Ref. [12]. The ZPL wavelength reads now $\lambda^{\mathrm{g}'}_{\mathrm{ZPL}} = (1279.4587 \pm 0.0003)$ nm. A detuning of $\delta'_{\mathrm{g}} = (-0.483 \pm 0.001)$ nm is therefore achieved. These results are shown in blue in Fig. S10b. Considerations analogous to how the plot in Fig. 3d is realized (see previous subsection) hold for Fig. 3e as well. Achieving the detuning δ'_{g} starting from δ_{g} is the result of several intermediate tuning steps, each corresponding to a different laser power used to tune the cavity. In more detail, we indicate with step 0 the initial situation where no tuning is performed, and with step 1 to step 5 the stages where powers of 250 μW, 320 μW, 400 μW, 450 μW and 550 μW, respectively, were used to burn the gas off the surface of the sample. Fig. S10b shows step 0 (corresponding to δ_{g}) and step 5 (corresponding to δ'_{g}) only. As mentioned above, this tuning mechanism blueshifts the cavity resonance, but may also spectrally shift the ZPL [12].

Examples of ZPL shifts are shown in Fig. S12a for the different tuning steps. From Lorentzian fits, we extract central wavelengths of (1279.277 ± 0.001) nm, (1279.239 ± 0.001) nm, (1279.167 ± 0.001) nm, (1279.0988 ± 0.0002) nm, (1279.126 ± 0.002) nm, (1279.4587 ± 0.0003) nm for steps 0 to 5, respectively. In practice, these shifts affect the coupling of the emitter to the cavity, whose resonance always blueshifts at each step. Fitting the cavity data with the same procedure described above, we extract central wavelengths of (1279.354 ± 0.002) nm,

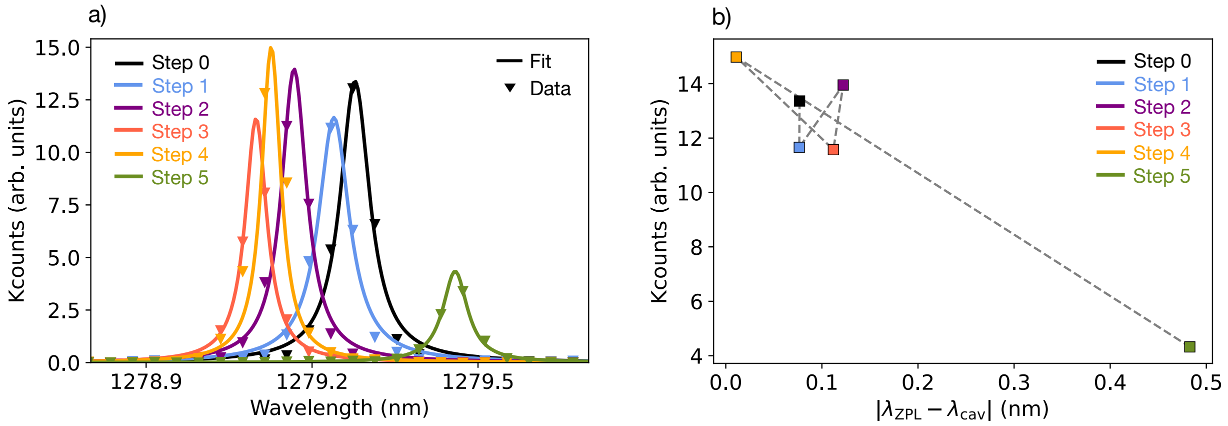

Figure S12. **Gas tuning steps.** a) Emitter's spectra measured at different gas-tuning steps. Step 0 corresponds to the initial condition, where no tuning is applied. Steps 1 to 5 correspond to different tuning steps at increasing laser powers. A shift in the ZPL wavelength is clearly visible. b) Counts versus relative shift between cavity resonance and emitter's ZPL. The first five points do not show a significant change in the ZPL intensity, due to the fact that the ZPL experiences a spectral shift. The non-monotonic behaviour of the counts when increasing the relative shift may be explained with the effect played by the additional FP resonances in the cavity profiles. We highlight that at step 4 the cavity and emitter are almost perfectly spectrally aligned, which results in the observation of maximum counts. In the last step, the cavity and emitter shift significantly in opposite directions. This decoupling results in a reduction of the ZPL intensity. Error bars are smaller than the markers. The gray dashed line is a guide to the eye.

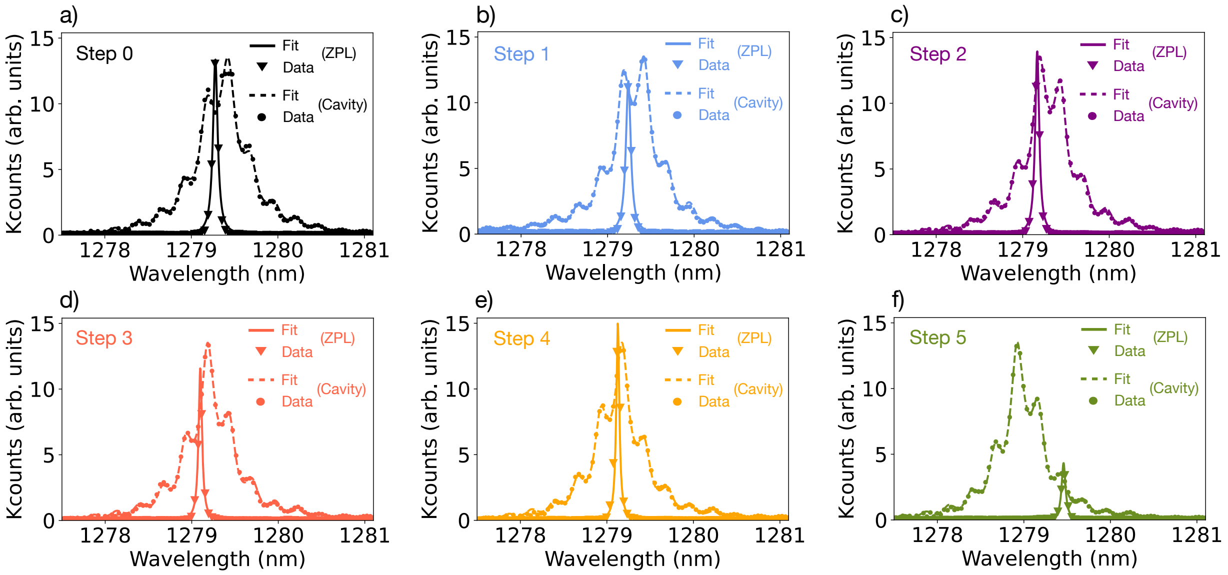

Figure S13. **Emitter and cavity's spectra at different tuning steps.** Cavity profiles (dashed lines) and ZPLs (solid lines) measured at a) step 0 to f) step 5. A clear relative shift of both the cavity resonance and the ZPL is visible when comparing e.g. step 4 and step 5. All cavity profiles are normalized to the same value for better visualization.

(1279.316±0.001) nm, (1279.290±0.001) nm, (1279.211±0.002) nm, (1279.137±0.002) nm, (1278.976±0.001) nm for steps 0 to 5, respectively. Fig. S12b shows the counts in function of the relative shift between the cavity resonance and the ZPL wavelength. From step 0 to 4, this shift stays small, which results in the observation of relatively similar counts (with step 4 showing maximum coupling). One would expect a monotonic behaviour when increasing the relative spectral shift. However, small variations in the intensity may be attributed to the FP oscillations, which may play a role in enhancing or de-enhancing the emission. The FP cavity profiles and ZPL spectra for each tuning step are reported in Fig. S13. There, it is visible that the emission at e.g. step 0 corresponds to a dip in the cavity profile. This means that, in principle, one would expect higher ZPL intensity in the ideal case without FP effects. Similarly, the emission in step 2 coincides with a maximum FP oscillation, meaning that its intensity would be lower in the ideal case. Similar conclusions can be drawn about all other steps. This would explain the non-monotonic behaviour of the counts in Fig. S12b. Additionally, beam repositioning after each tuning step might introduce minor additional errors to the measurements. At step 5, the cavity and emitter shift significantly in opposite directions, thereby effectively reducing their coupling. This results in a significant decrease in the ZPL intensity, as visible in Fig. S12b. As discussed in the main text, alternative approaches to (de)couple the emitter from the cavity may be based on electric field tuning [15] or mechanical strain [16].

7. Derivation of quantum efficiency

In the following, we derive the relevant rates from first principles and use them to estimate the G-center quantum efficiency of our system. We assume that 1) the cavity detuning does not significantly affect the Purcell factor of non-ZPL radiative phenomena (part of γ_0), and that 2) there is a negligible contribution of other radiative modes in our measurements due to our 0.1 nm narrow filtering of the ZPL. Under these assumptions, we write the total emission rate $1/\tau$ and the collected photon flux ϕ for both the on- and off-resonance cases as

$$\frac{1}{\tau_{\text{on}}} = (\alpha + F_{\text{P}})\gamma_{\text{R}} + \gamma_0, \tag{S8}$$

$$\frac{1}{\tau_{\text{off}}} = \gamma_{\text{R}} + \gamma_0, \tag{S9}$$

$$\phi_{\text{on}} = \eta\gamma_{\text{R}}(\alpha + F_{\text{P}}), \tag{S10}$$

$$\phi_{\text{off}} = \eta\gamma_{\text{R}}, \tag{S11}$$

with F_{P} the Purcell factor and $\eta = 0.7$ the coupling efficiency into the wanted mode extracted from our simulations in Fig. 2 in the main text. As already mentioned in the main text, γ_{R} is enhanced by a factor $(\alpha + F_{\text{P}}) = F_{\text{P}}^{*}$ when the emitter is placed in a cavity. This comes from considering F_{P}^{*} as a generalization of the originally formulated Purcell factor to the case of arbitrary photonic nanostructures [17]. In this case, F_{P}^{*} includes contributions from the cavity mode emission as well as from the emission from modes outside the cavity [17, 18]. This means that it can be written as $F_{\text{P}}^{*} = F_{\text{P}} + F_{\text{P}}^{\text{leak}}$, with $F_{\text{P}} = \frac{\gamma_{\text{R}}^{\text{cav}}}{\gamma_{\text{R}}}$ and $F_{\text{P}}^{\text{leak}} = \frac{\gamma_{\text{R}}^{\text{leak}}}{\gamma_{\text{R}}}$, where $\gamma_{\text{R}}^{\text{cav}}$ is the decay rate into the cavity mode and $\gamma_{\text{R}}^{\text{leak}}$ the decay rate into all other non guided modes. By defining $F_{\text{P}}^{\text{leak}} \equiv \alpha$, we can rewrite the generalized Purcell factor as $(\alpha + F_{\text{P}})$. The factor α depends on the specific cavity type and geometry.

The quantum efficiency QE is defined as

$$\text{QE} = \frac{\gamma_{\text{R}}}{\gamma_{\text{R}} + \gamma_0}\frac{1}{F_{\text{DW}}}, \tag{S12}$$

where F_{DW} is the Debye-Waller factor, i.e. the fraction of the PL intensity emitted in the ZPL, and is extracted from the literature. Extracting γ_0 from the equation above and substituting it in the ratio between Eqs. S8 and S9, that is $\tau_{\text{off}}/\tau_{\text{on}}$, we find an expression for $\tau_{\text{off}}/\tau_{\text{on}}$ as a function of QE:

$$\frac{\tau_{\text{off}}}{\tau_{\text{on}}} = 1 + \text{QE}F_{\text{DW}}(\alpha + F_{\text{P}} - 1). \tag{S13}$$

Following the procedure in Ref. [19], given that in our measurements the lifetimes remain constant within the error, we can define a bound on our QE by setting a threshold for the detection of the longest lifetime allowed by our off-resonance standard deviation $\tau_{\text{off-th}} = \tau_{\text{off}} - 3\sigma_{\text{off}}$. As the on- and off-resonance lifetimes do not differ significantly, it holds that $\tau_{\text{on}} > \tau_{\text{off-th}}$. Therefore, we can derive an upper bound for the QE:

$$\text{QE} < \frac{\frac{\tau_{\text{off}}}{\tau_{\text{off-th}}} - 1}{F_{\text{DW}}(\alpha + F_{\text{P}} - 1)}. \tag{S14}$$

To calculate QE from Eq. S14, an estimate of the Purcell factor F_{P} is needed. We can derive F_{P} from the ratio between the photon fluxes ϕ_{on} and ϕ_{off} in Eqs. S10 and S11:

$$\frac{\phi_{\text{on}}}{\phi_{\text{off}}} = \alpha + F_{\text{P}}, \tag{S15}$$

and thus find

$$F_{\text{P}} = \frac{\phi_{\text{on}}}{\phi_{\text{off}}} - \alpha. \tag{S16}$$

Deriving the Purcell factor F_{P} from Eq. S16 using our measured photon fluxes and substituting it in S14, we find

$$\text{QE} < \frac{\frac{\tau_{\text{off}}}{\tau_{\text{off-th}}} - 1}{F_{\text{DW}}(\frac{\Phi_{\text{on}}}{\Phi_{\text{off}}} - 1)}, \tag{S17}$$

which we use to estimate the QE to be bounded by (18±1)% for a measured off-resonance lifetime value of $\tau_{\text{off}} = (6.09 \pm 0.25)$ ns. We note that the bound on the QE is independent of the factor α dictating the radiative rate enhancement. The uncertainty on the bound is derived from propagated Poissonian statistics of the measured counts.

8. Comparison to the G-center literature

Supplementary Table 1 summarizes the reported literature on single emitters in silicon identified as G-centers in terms of their ZPL, estimated QE, $g^{(2)}(0)$, and excited state lifetimes. Even though a direct comparison of the efficiencies among these works is challenging due to different experimental parameters such as N.A. of the objective, filtering apparatus, and coupling and detection efficiency, we can qualitatively correct for the detection efficiency, when reported, and extract an indicative value of the count rates at saturation. Most of the cited works reporting saturation measurements on single G-centers would measure rates well below ~ 100 Kcounts/s. In our case, we would measure ~ 200 Kcounts/s instead. While this is just a qualitative estimate, we can conclude that our ZPL enhancement leads to notably bright single-photon emission. Supplementary Table 1 also includes a recent report on cavity-coupled ensembles [19]. What reported in the table aligns with the hypothesis, originally brought forward by Ref. [20], that the community is likely studying two different defects. The first one, which we label G_a [8, 12, 19–22], aligns with the original G-center ensembles, and features a ZPL at around 1278 nm with a narrow inhomogeneous linewidth, a lower QE, and a short excited state lifetime. The second one, G_b [7, 23], features a blue-shifted ZPL at 1270 nm with a high QE and a longer excited-state lifetime. We also point out that Ref. [23] reports a lifetime shortening from 54 ns down to 7 ns when reducing the coupling between the cavity and emitter. The shortened lifetime value is comparable to our measured values even in the absence of lifetime reduction.

Although our results suggest the possibility of two different artificial atom systems, the reported differences may still be due to a different host material, measurement setups, or fabrication protocols.

Reference	ZPL (nm)	QE (%)	$g^{(2)}(0)$	τ (ns)	τ^{cav} (ns)	Q/V_{cav} $(\lambda/n)^{-3}$	Notes
Redjem et al. [7]	1270.0 ± 9.1	~ 50*	0.3	35.8 ± 0.2			29 emitters
Hollenbach et al. [8]	1278.3 ± 0.1		$0.07 \pm 0.04^{\dagger}$	3.8			12 µm SOI, 12 emitters
Baron et al. [20]	1279		~ 0.3	4.5			1 emitter measured
Hollenbach et al. [21]	1278		0.36 ± 0.06	10.0			1 emitter measured
Prabhu et al. [12]	1278.7 ± 1.1	>1*	0.38 ± 0.08	8.3 ± 0.7			Waveguide, 37 emitters
Komza et al. [22]	1278	>2*	0.15 ± 0.02	4.6			Waveguide, 1 emitter
Redjem et al. [23]	1275	~ 1	0.30 ± 0.07	33.3	6.7	4862	L3 cavity, 1 emitter
Lefaucher et al. [19]	1279	<10**		5.6 ± 0.1	5.6 ± 0.1	417	Ensembles, static rings
**This work**	1279	<18	$0.03 \pm ^{0.07}_{0.03}$	6.1 ± 0.3	5.50 ± 0.05	> 3725	Opt. L3 cavity, 1 emitter

Supplementary Table 1. **Comparison of measured properties for the reported G-centers in the literature.** All the listed references other than Lefaucher et al. [19] report single-photon emitters. If several emitters are listed in Notes, the reported error is the statistical standard deviation, otherwise it is the measurement/fit error. *Estimated from photon counts and setup loss. **Extracted from ensembles and static cavities. † Background corrected.

[1] Andreani, L. C. & Gerace, D. Photonic-crystal slabs with a triangular lattice of triangular holes investigated using a guided-mode expansion method. *Physical Review B - Condensed Matter and Materials Physics* **73**, 1–16 (2006).
[2] Minkov, M. *et al.* Inverse design of photonic crystals through automatic differentiation. *Acs Photonics* **7**, 1729–1741 (2020).
[3] Minkov, M., Savona, V. & Gerace, D. Photonic crystal slab cavity simultaneously optimized for ultra-high q/v and vertical radiation coupling. *Applied Physics Letters* **111** (2017).
[4] Minkov, M. & Savona, V. Automated optimization of photonic crystal slab cavities. *Scientific reports* **4**, 5124 (2014).
[5] Panuski, C. L. *et al.* A full degree-of-freedom spatiotemporal light modulator. *Nature Photonics* **16**, 834–842 (2022).
[6] Altug, H. & Vucković, J. Polarization control and sensing with two-dimensional coupled photonic crystal microcavity arrays. *Optics Letters* **30**, 982–984 (2005).
[7] Redjem, W. *et al.* Single artificial atoms in silicon emitting at telecom wavelengths. *Nature Electronics* **3**, 738–743 (2020).
[8] Hollenbach, M., Berencén, Y., Kentsch, U., Helm, M. & Astakhov, G. V. Engineering telecom single-photon emitters in silicon for scalable quantum photonics. *Optics Express* **28**, 26111–26121 (2020).
[9] Beveratos, A., Brouri, R., Poizat, J.-P. & Grangier, P. Bunching and antibunching from single nv color centers in diamond. In *Quantum Communication, Computing, and Measurement 3*, 261–267 (Springer, 2002).
[10] Lin, Z., Schweickert, L., Gyger, S., Jöns, K. D. & Zwiller, V. Efficient and versatile toolbox for analysis of time-tagged measurements. *Journal of Instrumentation* **16**, T08016 (2021).
[11] Udvarhelyi, P., Somogyi, B., Thiering, G. & Gali, A. Identification of a Telecom Wavelength Single Photon Emitter in Silicon. *Physical Review Letters* **127**, 196402 (2021).
[12] Prabhu, M. *et al.* Individually addressable and spectrally programmable artificial atoms in silicon photonics. *Nature Communications* **14**, 2380 (2023).
[13] Warren, S. G. Optical constants of carbon dioxide ice. *Applied Optics* **25**, 2650–2674 (1986).
[14] Ismail, N., Kores, C. C., Geskus, D. & Pollnau, M. Fabry-Pérot resonator: Spectral line shapes, generic and related Airy distributions, linewidths, finesses, and performance at low or frequency-dependent reflectivity. *Optics Express* **24**, 16366–16389 (2016).
[15] Anderson, C. P. *et al.* Electrical and optical control of single spins integrated in scalable semiconductor devices. *Science* **366**, 1225–1230 (2019).
[16] Wan, N. H. *et al.* Large-scale integration of artificial atoms in hybrid photonic circuits. *Nature* **583**, 226–231 (2020).
[17] Lodahl, P., Mahmoodian, S. & Stobbe, S. Interfacing single photons and single quantum dots with photonic nanostructures. *Reviews of Modern Physics* **87**, 347 (2015).
[18] Faraon, A., Barclay, P. E., Santori, C., Fu, K.-M. C. & Beausoleil, R. G. Resonant enhancement of the zero-phonon emission from a colour centre in a diamond cavity. *Nature Photonics* **5**, 301–305 (2011).
[19] Lefaucher, B. *et al.* Cavity-enhanced zero-phonon emission from an ensemble of g centers in a silicon-on-insulator microring. *Applied Physics Letters* **122**, 061109 (2023).
[20] Baron, Y. *et al.* Single G centers in silicon fabricated by co-implantation with carbon and proton. *Applied Physics Letters* **121**, 084003 (2022).
[21] Hollenbach, M. *et al.* Wafer-scale nanofabrication of telecom single-photon emitters in silicon. *Nature Communications* **13**, 7683 (2022).
[22] Komza, L. *et al.* Indistinguishable photons from an artificial atom in silicon photonics. *arXiv preprint arXiv:2211.09305* (2022).
[23] Redjem, W. *et al.* All-silicon quantum light source by embedding an atomic emissive center in a nanophotonic cavity. *Nature Communications* **14**, 3321 (2023).
